# Users’ Adoption of Mental Health Apps: Examining the Impact of Information Cues

**DOI:** 10.2196/mhealth.6827

**Published:** 2017-06-28

**Authors:** Hsiao-Ying Huang, Masooda Bashir

**Affiliations:** ^1^ Illinois Informatics Institute University of Illinois at Urbana-Champaign Urbana, IL United States; ^2^ School of Information Sciences University of Illinois at Urbana-Champaign Champaign, IL United States

**Keywords:** user interaction design, recommendation system, mobile app search, mental health, anxiety

## Abstract

**Background:**

Numerous mental health apps have been developed and made available to users on the current app market. Users may find it difficult and overwhelming to select apps from the hundreds of choices that are available in the app marketplace. Clarifying what information cues may impact a user’s selection and adoption of mental health apps is now a critical and pressing issue.

**Objective:**

The aim of this study was to investigate the impact of information cues on users’ adoption of anxiety apps using observational data from the Android app market.

**Methods:**

A systematic search of anxiety apps was conducted on the Android app store by using keywords search. The title and metadata information of a total of 274 apps that met our criteria were collected and analyzed. Three trained researchers recorded the app rankings from the search results page on different dates and Web browsers.

**Results:**

Our results show that ratings (*r*=.56, *P*<.001) and reviews (*r*=.39, *P*<.001) have significant positive correlations with the number of installs, and app prices have significant negative correlations with installs (*r*=−.36). The results also reveal that lower-priced apps have higher ratings (*r*=−.23, *P*<.001) and a greater number of app permission requests (*r*=.18, *P*=.002) from the device. For app titles, we found that apps with titles related to symptoms have significantly lower installs than apps with titles that are not related to symptoms (*P*<.001).

**Conclusions:**

This study revealed a relationship between information cues and users’ adoption of mental health apps by analyzing observational data. As the first of its kind, we found impactful indicators for mental health app adoptions. We also discovered a labeling effect of app titles that could hinder mental health app adoptions and which may provide insight for future designs of mental health apps and their search mechanisms.

## Introduction

### Background

Mental disorders are a challenging public health issue. This is due to the high impact of these disorders on people, which limit their participation in all aspects of personal life, family life, and society. Mental disorders affect approximately one in four adults across the world at some point during their lifetime [1]. The high prevalence of mental disorders reveals a high demand for timely mental health care and intervention for people with mental disorders. However, the enormous cost of mental health care, the shortage of mental health professionals, and the barriers to accessibility make both diagnosis and treatment delayed or unavailable [1-3]. Therefore, how to provide affordable, in time, and accessible mental health care for those in need has become a critical and urgent issue.

With the rapid development of technology, the computing capacity of mobile technologies has advanced to the point that today’s mobile devices function like handheld computers and are highly integrated into our daily lives. According to surveys [4,5], over two-thirds (64%) of US adults own a mobile phone, and users, on average, check their phones 46 times a day. The prevalent ownership and use of mobile devices make mobile apps a promising venue with which to engage users into beneficial activities or therapeutic sessions in the context of mental health [6,7]. For instance, many mental health apps with self-monitoring mechanisms enable users to track their moods and emotions over time [8]. The personal use of mental health apps also provides confidentiality for users’ engagement, which may further encourage their adoption by young people and users who have a high sense of autonomy for seeking self-help [9]. With the advantage of continuous and ubiquitous access, mobile apps have the potential to decrease barriers for help-seeking and make therapeutic activities more accessible and less stigmatic [6,10-12].

Although mobile apps enhance the deployment of mental health interventions, there are still significant challenges in increasing their adoption and incorporating them into users’ daily lives in the real world. Thus, understanding users’ adoption of mental health apps becomes an important step toward designing and utilizing them as effective intervention approaches.

### Challenges in Mental Health App Adoptions

Mental health apps can encompass various functions ranging from guiding recovery for mental disorders to encouraging beneficial behaviors for improving emotional health [13,14]. For example, many mental health apps can assist clinical practice, engage real-time communication, or provide psychoeducation [15]. However, the adoption of mental health apps is rather distinctive from other types of apps because of its sensitive nature. The sensitivity of mental health issues can be attributed to the long-existing social stigma, which is the most common reason given for hindering people seeking mental health care or support [16,17]. Previous research has found that avoiding a social stigma becomes a significant reason for young people to use mental health apps [18]. Nevertheless, many available mental health apps target specific users and label them by diagnosis [14] that not only may exacerbate the stigma [6,8,14] but also affect users’ adoption of mental health apps.

As there is no clear guideline, regulations (eg, Health Insurance Portability and Accountability Act [HIPPA] or Food and Drug Administration [FDA]), or recommendation for users to select mental health apps, another challenge in mental health app adoption is that users may find it difficult and overwhelming to select the appropriate app from hundreds of choices available on the app market [19]. For instance, while browsing apps on the Android app store, users can only filter apps by rating or price. The filtering mechanism on the Android app store is limited. If a user wants to find an app to help alleviate her or his anxiety, she or he may need to go through all of the apps on a search results page. Another option for users is to randomly select an app, which may not correspond to the user’s needs. With more and more mental health apps available on the market, a critical and pressing issue is how to help users select and identify the “right” app for their mental health wellness. However, it is critical to understand the adoption of mental health app from the users’ perspective before designing reliable mechanisms to assist users’ app adoption.

### App Adoption as a Heuristic Process

App adoption can be regarded as a selection process in a computer-mediated context where users make their decisions by relying on a variety of information cues [20,21]. While making decisions, individuals often use a heuristic approach to process information instead of a systematic approach due to the human’s bounded rationality [22-24]. Heuristics are “process-oriented strategies” that allow individuals to make decisions in a faster and more frugal way by reducing cognitive efforts [24,25]. That is, when utilizing a heuristic approach, individuals are inclined to examine and integrate fewer information cues and simplify their weighing principles for cues [26].

In the selection process, heuristics comprise three stages: (1) searching, (2) stopping, and (3) deciding [23,25]. For example, when selecting an app, users may search for app titles that they recognize and stop the search once they categorize the available apps into either recognized or unrecognized titles. However, individuals may rely on multiple information cues rather than just a single cue during a heuristic process [27]. Thus, the consideration of multiple effects of information cues on users’ app adoption becomes important.

Previous studies have identified a variety of information cues that can affect users’ selection in app adoption, including prices, ratings, reviews, rankings, installs, titles, descriptions, functions, and privacy issues of apps [15,20,28-36]. A study by Dogruel et al [20], which is most relevant to ours, further points out that when users know what type of apps they need, they often employ the simple “take the first” heuristic approach, which is predominantly influenced by apps’ titles and crowdsourcing-based cues such as ratings and rankings of apps. Users only seek additional information (eg, reviews and functions) when they are uncertain about rating and ranking cues [20].

Existing research has indicated several influential cues involved in the process of app selection and adoption. However, the literature about how users select and adopt mental health apps is still rather scarce. As users’ app adoption varies by the kinds of apps, we still have no knowledge about the type of information cues that have relational impact on users’ adoption of mental health apps. Considering the sensitivity of the mental health context, we are curious whether predominant cues (eg, apps titles, ratings, and rankings) indicated by previous studies remain influential in app adoption. To the best of our knowledge, this study is the first work focusing on examining the relationship between information cues and mental health app adoptions.

**Figure 1:**
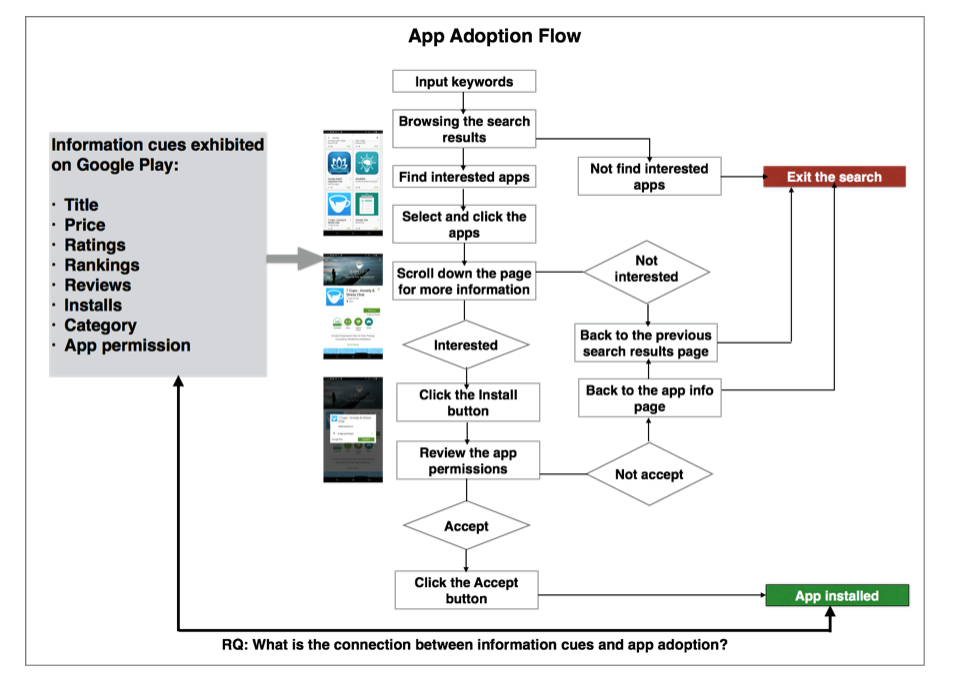
Research framework.

### Research Framework

The aim of this study was to investigate how exhibited information cues in an app store are related to mental health app adoptions. As mental health is a realm too broad to investigate, we focus in particular on one mental health condition: anxiety, which is also one of the most common mental health issues among US adults [37]. In addition, the delivery of psychological intervention via mobile devices is well suited for anxiety disorders because it allows users to receive in-time treatment during their daily routine, which makes anxiety disorders an ideal research topic.

We selected Android app store, Google Play, as our research site because it is currently one of the leading app marketplaces [38]. As exhibited in Figure 1, the anxiety app adoption flow starts from the keyword search. Users input the keyword and get the first search result page that mainly displays apps’ titles, ratings, and prices. If users are interested in one of the anxiety apps, they can click the app for more detail, such as the number of installs, reviews, and descriptions of the apps. After browsing the information, users can decide either to install the app or go back to their search results page for more options. If users decide to install the anxiety app, the permission consent dialogue will pop up to notify users what permissions are requested by the app. Apps will be installed if the users accept the app permission request.

As pointed out by previous studies [20,27], users’ app adoption is affected by multiple information cues. However, this type of multiple effect is difficult to measure or observe directly from the users’ self-report because of bounded rationality and self-bias. Therefore, we propose to use the observational data of apps for examining the multiple effects of information cues on app adoption instead of using users’ self-reported data. Based on prior research, we examined 8 types of information cues exhibited on Google Play, including titles, prices, ratings, reviews, rankings, installs, category, and app permission. We conducted several statistical analyses to explore the connections between these information cues and anxiety app adoptions, which are described in the Methods section.

## Methods

### Anxiety App Search and Selection

To simulate the users’ app search process, we used keyword search strategies to identify apps that most likely would be adopted by users seeking anxiety-related apps, which is similar to the approach employed by Ramo et al [39]. According to the Diagnostic and Statistical Manual of Mental Disorders, fifth edition (DSM-5) [40], we first identified 3 main keywords related to anxiety disorders, including anxiety, fear, and avoidance. Each term reported 250 results on Google Play. We decided to drop the term “avoidance” because its search results did not report anxiety-related apps. In order to identify other potential keywords, a search for the word “anxiety” was performed at the website UrbanDictionary.com. A total of 27 commonly used terms were listed, and two of the words most compatible with anxiety and fear, “anxious” and “worry,” were selected. Four keywords were used in our final search terms on Google Play, including anxiety, anxious, fear, and worry. We used “anxiety” as our primary search term and the other three keywords for supplementary searches.

**Figure 2:**
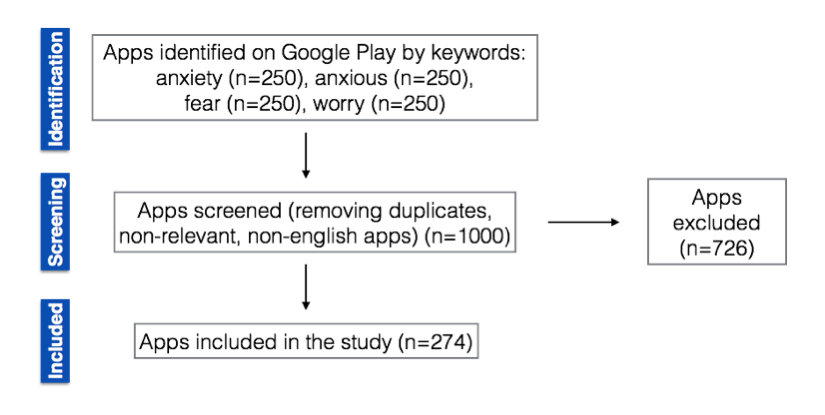
Systematic search of anxiety-related apps on Google Play.

We conducted a 2-phase app search. The first app search using these keywords was conducted on Google Play from July to September 2016. Researchers collected metadata information for all of the apps and selected the anxiety-related apps based on the apps’ descriptions. After selection, a second round of app searches by keywords was conducted on October 7, 2016, by 3 researchers. Twenty-four new apps were found and 14 apps no longer existed. A total of 274 apps were selected for analysis. Figure 2 displays the search process for anxiety apps on Google Play.

### Information Cues of Anxiety Apps on the Android App Store

#### Metadata as Information Cues

The search result of anxiety apps on Google Play provides the users different metadata information cues. According to Figure 1, we collected 8 types of metadata cues including (1) prices, (2) ratings, (3) reviews, (4) installs, (5) categories, (6) permissions, (7) ranking, and (8) title. We reassigned the number to the installs because we could only access the approximate range of installs on Google Play, instead of the exact number. Based on the range of categories, the number of installs ranges from level 1 (<10) to level 12 (>1,000,000). We want to note that installs, ratings, and reviews are represented as indicators for the adoption of apps.

#### App Ranking on Search Results Page

The search ranking of results has been considered as an influential factor on users’ selection [41]. Our assumption is that the more popular the app is, the higher search ranking it has. However, the app ranking of search results on Google Play is defined by algorithm, which may customize the ranking of apps based on individuals’ preferences. In order to identify the average mean ranking for each app, 3 researchers individually searched apps by keywords and recorded their rankings from October 7 to October 11, 2016. Considering that the size of a mobile screen is limited and not easy to view the ranking of all apps, we recorded the ranking on a Web browser instead of a mobile device.

In addition, to examine the stability of app ranking produced by the 3 researchers, we calculated the change of app ranking and its variability. As exhibited in Table 1, the variability of app ranking between researcher A and B is smaller than other comparisons. We also conducted paired samples *t* test to compare the mean difference of the change of app ranking among researchers. The results found no significant difference, indicating that the app ranking reported by researchers is fairly similar.

**Table 1:** Change of app ranking among researchers.

Statistics of app ranking change	Change 1^a^	Change 2^b^	Change 3^c^
Mean	−0.04	−1.91	−1.93
Median	0.00	0.00	0.00
Standard deviation	28.89	43.57	39.05

^a^Change 1 denotes the difference of app ranking between researcher A and B.

^b^Change 2 denotes the difference of app ranking between researcher B and C.

^c^Change 3 denotes the difference of app ranking between researcher A and C.

### Classifying Titles of Anxiety Apps

The title of an app gives users their first piece of information on what the app is about, which can further influence the users’ apps selection [29]. However, the functionality of apps, such as treatment, psychoeducation, and diagnosis, may not always appear in the title. Instead, they may emphasize specific disorders, symptoms, or activities. We are interested in whether anxiety apps tend to use certain terms in their titles. To classify the titles of anxiety apps, we adopted the recommendation proposed by [14] and generated six criteria from both clinical and nonclinical perspectives. The clinical criteria include anxiety disorders, symptoms, and treatments, and the nonclinical criteria are self-help approaches, mindfulness activities, and the self-tracking or management tool. We reviewed the title for each anxiety app to see if they are related to these six aspects. The details of the six criteria are exhibited in Textbox 1.

**Classifying criteria of anxiety app titles.**

Classifying criteria

- Specific type of anxiety disorders (eg, social anxiety disorder, generalized anxiety disorders, and panic attacks)
- Symptoms (eg, fear, anxiety, and worry)
- Treatments (eg, cognitive behavioral therapy [CBT], counseling, and therapy)
- Self-help approach
- Mindfulness activities (eg, breathing, meditation, and body scan)
- Self-tracking or management tools (eg, mood tracker or monitor, diary, and stress management)

### Data Analysis

To capture the connections and multiple effects of information cues on anxiety app adoptions, we employed different statistical techniques. We first examined the relationship among metadata information cues and app adoption by correlational analysis. We then tested the normality of each information cue using the Kolmogorov-Smirnov test and found that these variables were not normal distribution. Therefore, nonparametric approaches including the Kruskal-Wallis test and the Mann-Whitney test were adopted to examine the differences of app adoption by app categories and titles.

In order to test predictable effects of information cues on anxiety app adoptions, we applied a nonparametric regression technique known as generalized additive model (GAM) proposed by Hasite and Tibshirani [42]. The GAM framework assumes that the contribution of each predictor is additive, which is similar to the concept of linear regression that each variable is estimated independently. The dependent variable is an additive combination of arbitrary functions of predictors. This additive modeling technique captures the underlying predictive patterns of data by smooth functions, especially when the model has nonlinear effects [43]. In this study, the flexibility of GAM allows us to predict the nonlinear impact of each information cue on app adoption. We implemented GAM by the function gam() with regression splines in the R package “mgcv.”

## Results

### Overview of Anxiety Apps

#### Descriptive Statistics of Information Cues

**Figure 3:**
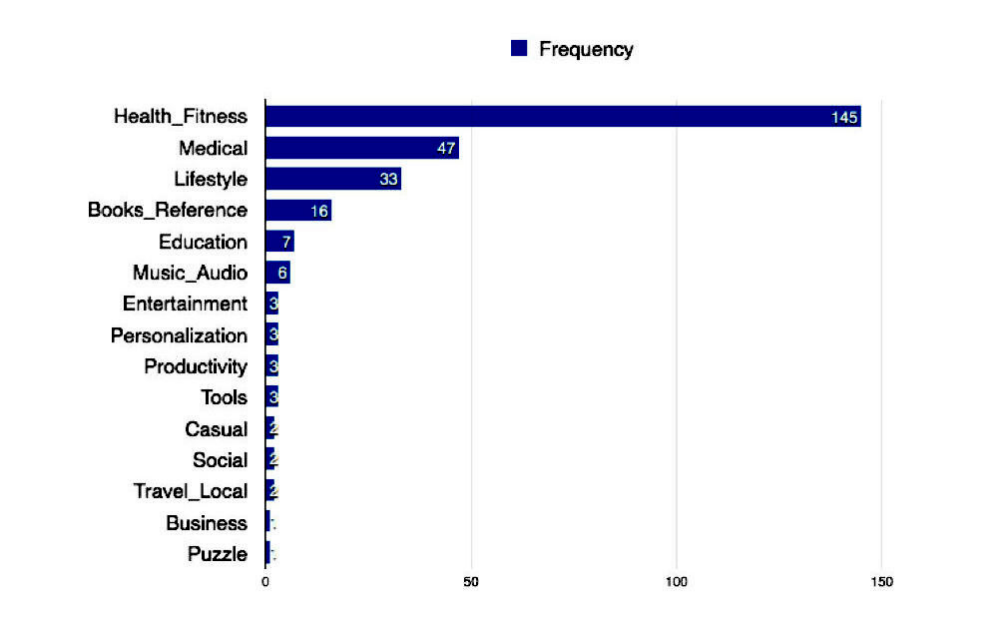
Anxiety apps in different categories on Google Play.

According to Table 2, the average price for anxiety apps on Google Play is US $0.81. The 3rd quartile price is US $0, which means that most apps are free. The median of app rating indicates that half of the apps have a rating higher than 4.1. Although the mean number of review is around 2686, the median shows that half of the apps have reviews lower than 35. The average install is 6.42, which is between 1000-5000. On average, anxiety apps request about 6 permissions from users’ mobile devices. In the included categories, 52.9% (145/274)of apps are in health and fitness, 17.2% (47/274) of apps are in medical, 12.0% (33/274) are in lifestyle, 5.8% (16/274) are in books and reference, and 12.0% (33/274) are in other categories (Figure 3).

**Table 2:** Descriptive statistics of anxiety apps on Google Play.

Descriptive statistics of metadata	Price	Rating	Review	Install	Permission	Ranking
Mean	0.81	3.43	2686.42	6.42	6.16	112.54
Standard deviation	2.09	1.61	11475.97	2.97	4.64	64.78
Minimum	0.00	0.00	0.00	1.00	0.00	1.00
1st Quartile	0.00	3.38	2.0	4.00	3.00	57.08
Median	0.00	4.10	35.50	6.00	5.00	111.00
3rd Quartile	0.00	4.40	568.2	8.75	9.00	157.83
Maximum	16.69	5.00	151870.0	12.00	37	246.67

#### Observations of Anxiety App Rankings

As exhibited in Table 2, the average app ranking is 112.54. Interestingly, the minimum and maximum app ranking is 1 and 246.67, respectively. This implies that an app is always at the top of the search results, but no app is always at the bottom of the search results. We further found that 7.7% (n=21) of apps appear to have the same ranking on the search results from all 3 researchers, and 34.3% (n=94) of the apps have the same ranking on the search results from 2 researchers, although 58% (n=159) of apps appear to have different rankings on the search results from all 3 researchers. These findings suggest that the ranking orders of anxiety apps may be personalized by algorithm.

#### Types of Titles Used by Anxiety Apps

**Figure 4:**
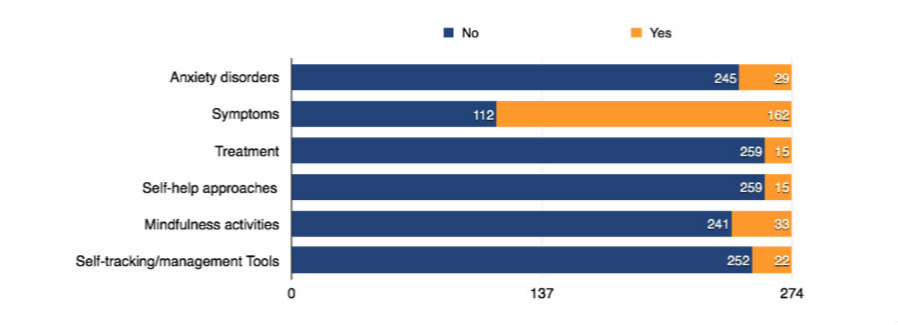
Number of app titles related to anxiety disorders, symptoms, treatment, self-help approaches, mindfulness activities, and self-tracking or management tools.

We found 211 app titles related to at least one of the six categories and 63 app titles not relevant to any of these 6 categories. Very few apps used clinical terms (eg, disorders and treatment) in their titles. As exhibited in Figure 4, only 10.6% of the apps associated their titles with anxiety disorders and 5.5% of the apps mentioned treatment in the titles. Over half of the apps (59.1%) used symptoms, but only 5.5% of the apps indicated a self-help approach in the title. 12% of the titles were related to mindfulness activities, and 8% were related to self-tracking or management tools.

### Metadata as Information Cues and Adoptions of Anxiety Apps

We examined the relationship between app adoptions and exhibited metadata information by correlational analysis. As exhibited in Figure 4, the ranking of apps have a significant positive correlation with app price, indicating that the higher price apps have, the lower ranking they have in the search results. The results also showed that the app ranking has a negative correlation with app review, rating, and installation. These results suggest that apps with a higher ranking also have more reviews, installs, and higher ratings.

**Figure 5:**
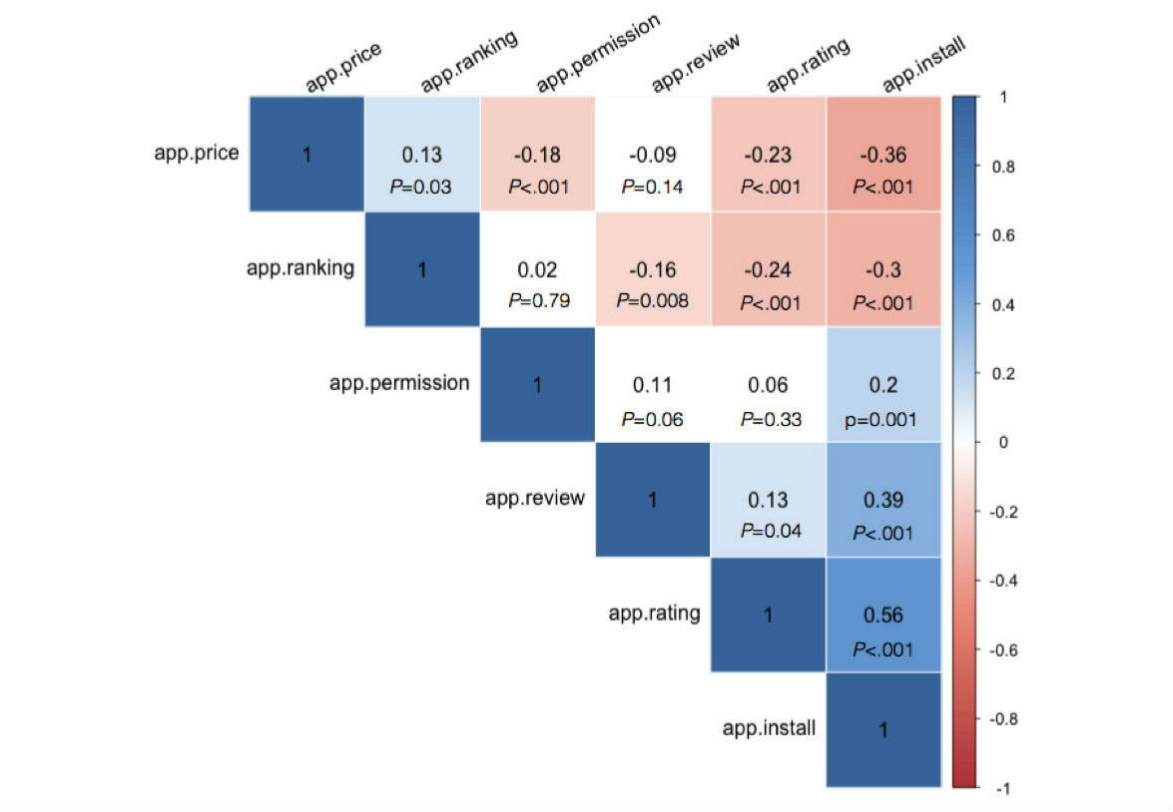
Correlational analysis of information cues and app adoption.

Another interesting result is that the total number of app permission requests positively correlates with installs. This suggests that apps requesting more app permissions from mobile devices have more installs. The results also show that apps with lower prices request more app permissions from the devices. Furthermore, the ratings and reviews of apps have significant positive correlations with the number of installs, and the price of apps has significant negative correlations with installs (Figure 5). This means that apps with higher ratings and reviews at lower prices have a higher number of installs. We want to note in particular that although these results have significant correlations, the coefficients are only at moderate or low level. It is also important to note that significant correlations only show the relationship between these observational cues that do not represent the cause and effect relations.

We applied the Kruskal-Wallis test to examine whether different categories of anxiety apps showed significant differences in ratings, reviews, and installs. As shown in Table 3, the results show the significant differences in price, rating, review, and install. According to the post-hoc test, apps in books and reference have significantly lower ratings (*P*=.05) and installs (*P*=.01) than apps in other categories. Also, apps in books and reference have a significantly lower amount of reviews than apps in health and fitness (*P*=.04) and others (*P*=.002). There is no significant difference among categories in app rankings.

**Table 3:** Kruskal-Wallis test for price, rating, review, install, and permission requests by apps category.

Category	Mean rank		
	Rating	Review	Install
Books and reference (N=16)	87.16	80.84	89.41
Health and fitness (N=145)	138.83	139.01	139.99
Lifestyle (N=33)	118.47	120.73	118.7
Medical (N=47)	147.73	136.18	134.81
Other (N=33)	153.05	169.66	167.82
Kurskal-Wallis chi-square (df^a^=4)	10.58 *P*=.03	15.227 *P*=.004	13.001 *P*=.01

^a^df: degrees of freedom.

### Predictable Effects of Information Cues on Adoptions of Anxiety Apps

We adopted GAM to predict the impact of 5 metadata information cues (price, rating, review, ranking, and permission) on adoptions of anxious apps. To select the smooth function of parameters for minimizing the prediction error, we used deviance and generalized cross validation score [44] as indicators. Deviance (D) represents the discrepancy between observations and fitted values, and generalized cross validation (GCV) estimates the expected fit of a model to a dataset. The smaller these scores are, the better fit the model has. Thus, we decided to smooth all variables because the model produces the lowest deviance (D=316.24) and GCV score (GCV=1.51). As shown in Table 4, the price, rating, and review have significant predictable effects on app adoption. Note that although ranking cue does not reach a significant level, it is still very close.

**Figure 6:**
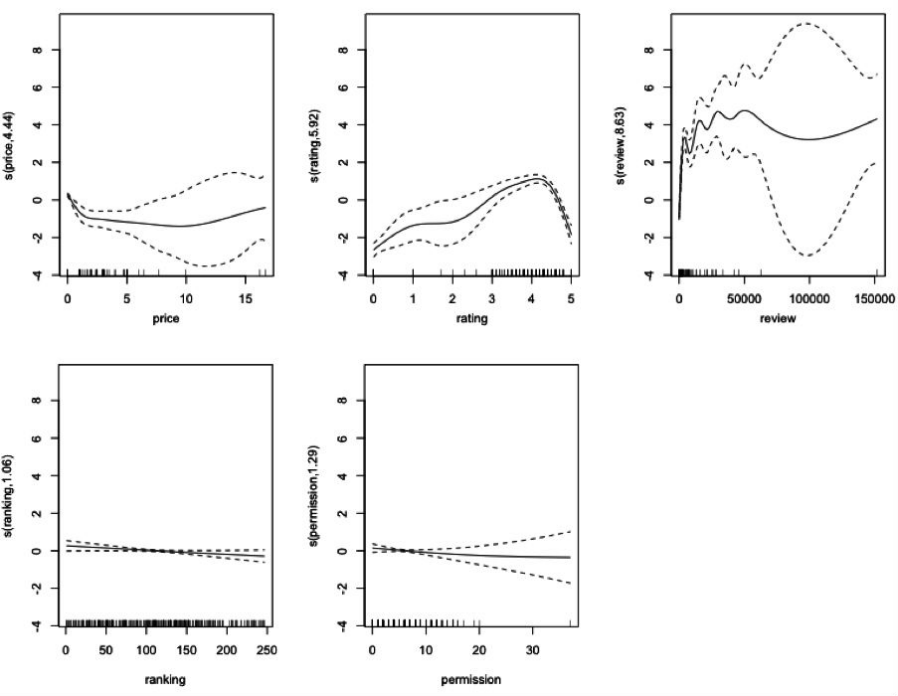
Generalized additive regression plots of information cues.

Figure 6 displays the direction of predictive impact on app adoption. We found that the expected number of app installs will decrease drastically at first when the price of the app increases. However, the decrease of expected installs becomes gradual while the price increases. This may suggest that when prices of apps are over a certain amount, the number of app installs will remain similar. For rating, the app installs will increase when the rating also increases until the rating reaches nearly 4.4. After this point, the number of installs will start decreasing as the rating increases. This also implies that the high rating apps may not always get high installs. Furthermore, the result shows that the app installs will increase when the number of reviews also increases. Yet, when the app review reaches approximately 50,000, the number of installs will slightly decrease until the number of reviews is over 100,000. For ranking, the higher ranking the app has, the more expected installs the app will have.

**Table 4:** Generalized additive model (GAM) of anxious app adoption.

Smooth (predictors)	Effective degrees of freedom	*F* value
Price	4.44	9.00 (*P*<.001)
Rating	5.92	44.36 (*P*<.001)
Review	8.63	40.34 (*P*<.001)
Ranking	1.06	3.42 (*P*=.06)
Permission	1.29	1.30 (*P*=.26)

### Titles as Information Cues and Adoptions of Anxiety Apps

An important function of the app title as an information cue is to inform users about the apps. We examined whether the linguistic cues in an app title are related to the users’ adoptions of anxiety apps. Considering that app installs, ratings, and reviews are not normally distributed, we applied the nonparametric Mann-Whitney test to examine the difference. Our results show that app titles using the terms relating to anxiety disorders have significantly fewer installs and reviews (Table 5). We also found that apps with titles relating to symptoms have significantly lower installs, ratings, and reviews than those without symptom-related titles. On the other hand, apps with titles relating to mindfulness activities have higher installs, ratings, and reviews than those without mindfulness-related titles. Overall, these results suggest that apps with titles not directly related to anxiety disorders or symptoms, but to mindfulness activities, are more adopted by users.

**Table 5:** Nonparametric Mann-Whitney test of installs, ratings, and reviews by types of titles.

Metadata information	Title	Mean rank: No (=0)	Mean rank: Yes (=1)	Mann-Whitney *U* (Significance)
**Install**	Anxiety disorders	141.73	95.59	2270.50 (*P*=.003)
	Symptoms	188.52	101.16	3245.50 (*P*<.001)
	Treatment	137.67	125.53	1763.00 (*P*=.56)
	Self-help	135.51	162.60	1551.00 (*P*=.19)
	Mindfulness	129.16	196.08	1965.50 (*P*<.001)
	Self-tracking tool	137.85	127.34	2548.50 (*P*=.55)
**Rating**	Anxiety disorders	138.86	116.71	2949.50 (*P*=.15)
	Symptoms	166.60	115.75	5594.00 (*P*<.001)
	Treatment	135.89	147.03	1769.50 (*P*=.59)
	Self-help	136.17	142.20	1842.00 (*P*=.77)
	Mindfulness	129.44	187.65	2255.50 (*P*<.001)
	Self-tracking tool	136.87	132.25	2656.50 (*P*=.79)
**Review**	Anxiety disorders	141.62	93.59	2279.00 (*P*=.002)
	Symptoms	190.88	99.01	2899.50 (*P*<.001)
	Treatment	137.12	125.90	1768.50 (*P*=.59)
	Self-help	135.28	157.43	1613.50 (*P*=.29)
	Mindfulness	128.87	191.73	2121.00 (*P*<.001)
	Self-tracking tool	137.02	130.61	2620.50 (*P*=.71)

## Discussion

### Principal Findings

#### Observation of Information Cues and Anxiety App Adoptions

##### App Price, Rating, and Review as Information Cues

One of our research questions was to examine the association with metadata information cues and anxiety app adoptions. Our findings suggest that prices, ratings, and reviews not only have significant correlations with adoptions of anxiety apps but also are impactful predictors on app adoptions. For instance, we found that price is a negative predictor of app adoptions. The lower-priced anxiety apps yield higher installs, which corroborate previous findings that most users tend to use apps with lower prices [29-31], even when it comes to mental health apps. Since mental health apps may pose negative influences on users, further examination on the quality of lower-priced mental health apps is needed.

Furthermore, the results indicate that the higher rating the anxiety app has, the more installs the app will have. However, interestingly, this positive relationship between rating and adoption is only predictable before the app rating reaches a certain point (Figure 6). The expected number of app installs will decrease when the app rating is over approximately 4.4. This finding further implies that some apps may have high ratings but do not always have high installs by users. A possible explanation is that users may consider multiple cues rather than a single cue when adopting the app [27]. For example, users may be inclined to select an app with high ratings and high reviews instead of an app with high ratings but low reviews.

Our analyses show that an app review can be a positive predictor of app install. An anxiety app will have more installs when it has more user reviews. Nevertheless, it is important to note that the positive prediction between review and adoption only exists initially. The relationship between review and adoption becomes dynamic when the number of reviews is over a certain point (Figure 6). Overall, as information cues, we found that app price, rating, and review can be important indicators when it comes to the adoption of anxiety apps. Also, the results of rating and review cues may further demonstrate the bandwagon effect on anxiety app adoptions [20,27]. Users may still follow or rely on other users’ adoptions to decide which apps to adopt.

##### App Permission and Category as Information Cues

Our findings indicate that anxiety apps requesting permissions from mobile devices have more adoptions by users, although app permission is not a significant predictor of adoption. We infer that apps with more permission requests may also provide more functions to users. In order to use apps, users may choose to trade off their long-term privacy for immediate gratification because of bounded rationality [45]. This may be the reason why anxiety apps with more permission requests are more adopted by users. Another possible explanation is that users may have less or no knowledge about app permissions; thereby, app permission may not be an important or useful cue for app adoption.

In terms of app category, our findings suggest that apps in books and reference have significantly lower installs, ratings, and reviews than apps in other categories. However, we cannot assert that users prefer anxiety apps in specific categories because apps in other categories may also show up in the search results from other terms, which can increase their adoption by users. Therefore, the app category may not be an effective information cue on app adoption. We suggest that the effect of app permission and category cues on app selection and adoption needs more investigation.

##### App Ranking as Information Cues

Our results indicate two important findings. First, app ranking generated by an algorithm can vary by individual. Second, apps with a higher ranking on the search results page also have more installs by users, although app ranking is not a significant predictor of app adoption in our model. We also found that anxiety apps with higher ratings and more reviews have higher rankings on the search results page. This indicates that app rating and review may be important factors in the design of an app search algorithm on Google Play.

#### Observation of Titles and Adoptions of Anxiety Apps

We further investigated influences of an apps’ title and discovered two interesting trends. Our results reveal that only a small fraction of anxiety apps use specific anxiety disorders and treatment in their titles. This finding is 2-fold. On the one hand, this may be a progressive sign for reducing the stigma and labeling for users; on the other hand, users may not easily find the apps with clinical information or assistance.

##### App Title Related to Disorders and Symptoms as Information Cues: Labeling Effect

Approximately 10% of anxiety apps use titles related to anxiety disorders, and 60% of apps have titles related to symptoms. The results show that apps with titles related to anxiety disorders and symptoms have lower adoptions and fewer reviews than others. Since app titles related to disorders or symptoms may label users with disorders or diagnoses [14,46], social or self-stigma of mental disorders may hinder users’ adoption of apps and decrease their motivation to provide app reviews. The disorder and symptom-related app titles as information cues may signal a stigma that prevents users’ adoptions.

##### App Title Related to Mindfulness as Information Cues: Positive Enhancement

Mindfulness is an approach to enhance self-awareness, openness, and acceptance to experience and to develop new perspectives on the context through focusing on the present moment [47-49]. Several studies have suggested that mindfulness is beneficial for personal recovery from mental disorders and to an individual’s positive well-being [36,50,51]. Interestingly, we discovered that anxiety apps with titles related to mindfulness activities have more installs, reviews, and higher ratings by users. Since app titles related to mindfulness activities (eg, breathing and meditation) signal providing a method to help users reduce their anxiety, users may perceive them to be more useful and applicable. In addition, mindfulness-related titles are not directly associated with disorders and symptoms; this reduces the labeling effect on users. In other words, anxiety apps with mindfulness-related titles signal positive enhancement that may further encourage users’ adoption.

#### Implications of Findings

Our findings can be applied to improving current design mechanisms of the app market for users’ selection and adoption of mental health apps. For example, app developers should avoid labeling effects when designing information cues for anxiety apps as suggested by previous research [14]. Considering the sensitivity of mental health issues, we suggest developers employ information cues that endorse positive enhancement to motivate users to adopt and engage with the apps.

From the observational data of anxiety apps, we learn that information cues with social influence may have significant impact on the adoption of apps. It seems that most users incline to “follow the crowd” when adopting anxiety apps. However, this follow-the-crowd strategy may mislead users to adopt an impractical or inappropriate app that may have harmful consequences. To help users select and adopt appropriate mental health apps, we suggest that developers design an information cue that signals the function or purpose of the apps on search results pages. This type of information cue may provide users with a better understanding of the apps and further assist users’ adoption of mental health apps.

### Limitations and Future Directions

In this study, we only examined anxiety apps on Google Play, which may limit our findings to a specific mental health context and app market. We recommend future studies apply the same approach to other mental health conditions and app markets and compare their results with this study. Although we found the correlational impact of information cues on app adoptions by using observational data of apps, we were unable to identify their cause and effect on app adoption. Therefore, we suggest future studies conduct empirical work based on the assumptive inferences we proposed in this study and investigate the cause and effects of information cues on users’ mental health app adoption.

### Conclusions

Mental health apps can be a powerful instrument for mental health care and intervention. How to design an app-searching system that can lead users to select the right apps for their mental well-being becomes a challenging issue. Since app adoption is a heuristic process, information cues play important roles. To clarify the impact of information cues on users’ app adoption, we examined the relationship between information cues and users’ app adoptions by using observational data in an app market. We discovered that app price, rating, and review are important indicators for anxiety app adoptions. On the other hand, the impact of app permission and category cues remain unclear. Most importantly, our findings revealed the importance of app titles on users’ selection and adoption of anxiety apps. Although there is still a long way to go for designing an effective search mechanism for mental health apps, our work demonstrates interesting phenomena and provides insight into the information cues and anxiety app adoptions, which will help provide better solutions for the future design of search mechanisms for mental health apps.

## Abbreviations

FDA: Food and Drug Administration
HIPAA: Health Insurance Portability and Accountability Act
